# Editorial: Tracing the cellular symphony: unraveling brain aging

**DOI:** 10.3389/fnagi.2026.2019461

**Published:** 2026-10-26

**Authors:** Elisa Mitiko Kawamoto, Shong Lau, Priya Balasubramanian

**Affiliations:** 1 University of São Paulo, São Paulo, Brazil; 2 ScienCell Research Laboratories, Carlsbad, CA, United States; 3 The University of Oklahoma Health Sciences, Oklahoma City, OK, United States

**Keywords:** aging, biomarkers, brain, memory, neurodegeneration

Aging leads to progressive alterations in cellular function across multiple organ systems, including the brain. Age-related molecular changes in metabolism, gene regulation, redox homeostasis, and neurotransmitter signaling can disrupt tissue integrity and contribute to structural changes, including blood-brain barrier disruption, white matter injury, and region-specific changes in brain volume. Recent advances in -omic technologies also shed light on cell-type-specific aging trajectories and how the coordinated interplay between multiple cell types in different brain regions contributes to functional decline manifested as cognitive impairment in aging and neurodegeneration in AD (Jin et al., 2025; Muralidharan et al., 2025). In this research topic, we bring together articles that utilize distinct approaches to characterize the aging brain at the molecular, structural, and functional levels, which will aid in distinguishing healthy aging from pathological states.

Identifying biomarkers may facilitate early diagnosis of CNS pathology. The earlier the diagnosis, the sooner the therapeutic intervention can begin. This is particularly true for age-related neurodegenerative diseases, as neuronal loss is often progressive and irreversible (Wilson et al., 2023). In the study by Choi et al., the authors investigated region-specific brain metabolite changes in 20 cognitively intact older adults aged 56–84 years using 3T proton magnetic resonance spectroscopy (1H-MRS). The findings from this study demonstrate that normal brain aging involves distinct, region-specific metabolic remodeling and highlight the potential of non**-**invasive ^1^H-MRS imaging for quantifying key metabolic alterations associated with aging and neurodegenerative diseases. In the study by Liu et al., using a combination of bibliometric analysis with machine learning approaches, the authors identified two selenium-associated genes, specificity protein 1 (SP1) and selenophosphate synthetase-2 (SEPHS2), that were altered in the brain during aging and may potentially serve as biomarkers for the aging brain.

Sex-based differences are an understudied area in the aging field. Although the literature presents some information regarding possible differences between men and women in terms of vulnerability to developing certain CNS-related diseases, there is still limited knowledge on how gender differences impact brain aging and, in turn, therapeutic strategies for age-related brain disorders. Jullienne et al. characterized normative brain aging by examining regional morphology and cerebrovascular architecture in male and female C57BL/6N mice of different ages using high-resolution MRI and vessel painting. Aging produced significant regional and sex-dependent changes in brain volume, with cortical volume declining in both sexes, while hippocampal regions remained relatively preserved. White matter volumes increased with age but more prominently in females. Interestingly, age or sex did not impact the cortical vasculature in this study. However, cerebrovascular pathology often precedes cognitive impairment in aging and neuronal loss in Alzheimer's disease (AD) (Barisano et al., 2022; Montagne et al., 2015). In particular, endothelial senescence and subsequent dysfunction contribute to cerebral hypoperfusion and blood-brain barrier leakage. Huang et al. investigated the therapeutic effects of Astragaloside IV (AS-IV) in mitigating endothelial senescence and AD pathology. AS-IV reduced hypoxia-reoxygenation-induced oxidative stress and endothelial senescence by modulating Notch1/VCAM-1 signaling and highlighted the therapeutic potential of AS-IV for treating vascular pathology in aging and AD.

Müller et al. reviewed the emerging advances in brain aging by focusing on the roles of coordinated interactions among glial cells, neurons, and vascular components in regulating tissue homeostasis during aging. The authors propose the concept of “cellular choreography” as a systems-level framework for understanding how these interconnected cellular populations collectively shape the aging brain. Importantly, the review highlights single-cell, spatial, longitudinal, and multi-omic approaches as powerful tools for defining cell-type- and region-specific aging trajectories, understanding altered intercellular communication, and identifying potential therapeutic targets to promote healthy brain aging. In another review article by Jackson et al., the authors outline the locus coeruleus-norepinephrine (LC-NE) system as a key regulator of gamma oscillations, linking novelty detection, attention, memory encoding, and cognitive resilience. The therapeutic effects of 40 Hz gamma stimulation are proposed to arise from the activation of LC-mediated neuromodulatory pathways that reduce neuroinflammation, enhance blood and glymphatic flow, promote synaptic plasticity and myelination, improving hippocampal-dependent memory.

Finally, motor dysfunction is one of the leading cause of falls and fractures, which culminates in hospitalization and loss of independence in the elderly population. Transcranial alternating current stimulation (tACS) has emerged as a noninvasive neuromodulation technique to ameliorate cognitive and motor dysfunction in aging and AD (Nissim et al., 2023). In this research topic, Zang et al. showed that 20 Hz tACS improved post-anesthesia motor performance in aged male mice. Further investigation is warranted to evaluate if tACS can also ameliorate age-related motor dysfunction in the awake state.
